# Pharmacological inhibition of perforin dampens CD8^+^ T cell-mediated beta cell destruction in autoimmune diabetes in mice

**DOI:** 10.1007/s00125-026-06773-8

**Published:** 2026-06-11

**Authors:** Prerak M. Trivedi, Tara Catterall, Vivien R. Sutton, Viki Moshovakis, Lorraine Elkerbout, Hedieh Akhlaghi, Julie A. Spicer, Louis Boon, Thomas W. H. Kay, Joseph A. Trapani, Helen E. Thomas

**Affiliations:** 1 St Vincent’s Institute of Medical Research, Fitzroy, VIC Australia; 2 Cancer Immunology Program, Peter MacCallum Cancer Centre, Melbourne, VIC Australia; 3 Sir Peter MacCallum Department of Oncology, The University of Melbourne, Parkville, VIC Australia; 4 Auckland Cancer Society Research Centre, Faculty of Medical and Health Sciences, University of Auckland, Auckland, New Zealand; 5 JJP Biologics, Warsaw, Poland; 6 Department of Medicine, St Vincent’s Hospital, The University of Melbourne, Fitzroy, VIC Australia

**Keywords:** Apoptosis, Autoimmunity, Beta cell, Cytotoxic T lymphocyte, Focused immunosuppressant drug, Granzyme, NOD mouse, Perforin, Type 1 diabetes

## Abstract

**Aims/hypothesis:**

CD8⁺ T cells are key mediators of beta cell destruction in type 1 diabetes, employing the perforin/granzyme and Fas/Fas ligand pathways. Evidence from our studies and others indicates that perforin plays a dominant role in CD8⁺ T cell mediated beta cell destruction in non-obese diabetic (NOD) mice. As perforin is indispensable for delivering pro-apoptotic granzymes into the cytosol of target cells, pharmacological inhibition of perforin may potentially protect beta cells against CD8^+^ T cell cytotoxicity. Such a strategy will slow down beta cell loss and the progression to autoimmune diabetes. Small molecule perforin inhibitors recently becoming available provided an opportunity to test this hypothesis.

**Methods:**

The new small molecule perforin inhibitor SN34960 was evaluated for its ability to protect the beta cells of non-obese diabetic mice upon attack by autoreactive cytotoxic T lymphocytes, both in vitro and in vivo.

**Results:**

In vitro, perforin inhibitor SN34960 protected isolated pancreatic islets from CD8^+^ T cell-mediated destruction (*p*<0.001). In vivo, a single dose of SN34960 reduced the death of islet-specific glucose-6-phosphatase catalytic subunit-related protein (IGRP) peptide-loaded target cells by ~50% over 16 h compared with non-peptide-loaded control cells (*p*<0.05). Furthermore, prediabetic NOD mice treated with perforin inhibitor for 7 days had a reduced incidence of diabetes when autoimmune attack was accelerated by anti-PDL1 blockade (91.2% vs 58.4%, *p*<0.05). Pharmacological perforin inhibition with SN34960 did not alter the activation or differentiation status of pathogenic cytotoxic T lymphocyte infiltrating the pancreas; instead, its mode of action is specifically to block the membrane-disruptive and pro-apoptotic effects of perforin following its secretion into the synaptic cleft.

**Conclusions/interpretation:**

Our results suggest that pharmacological perforin inhibition may represent a novel approach to protect beta cells from destruction by autoreactive CD8^+^ T cells.

**Graphical Abstract:**

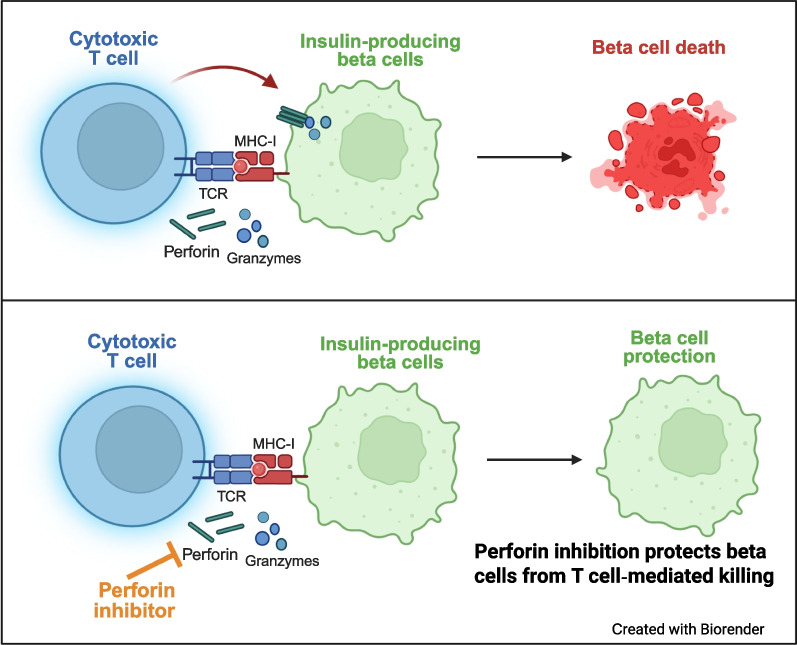

**Supplementary Information:**

The online version contains peer-reviewed but unedited supplementary material available at 10.1007/s00125-026-06773-8.


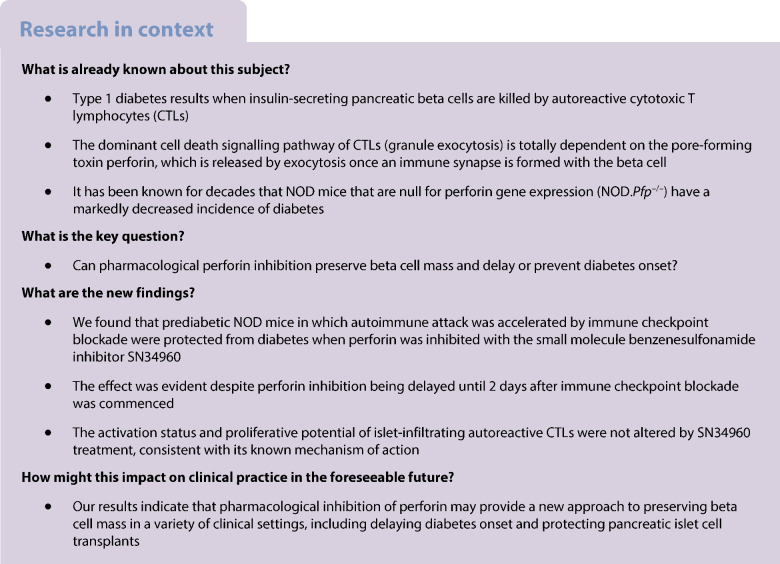


## Introduction

In humans with type 1 diabetes and in non-obese diabetic (NOD) mice, pathogenic CD8^+^ cytotoxic T lymphocytes (CTLs) kill beta cells when their T cell receptors (TCRs) engage with endogenous beta cell peptide antigens presented on MHC class I molecules. Upon stable synapse formation, secretory vesicles in the CTL migrate along microtubules to the site of cell–cell contact and release the pore-forming toxin perforin and granzyme serine proteases into the synaptic cleft via exocytosis [1]. Perforin monomers oligomerise into pores in the target cell plasma membrane, through which diverse granzymes diffuse to initiate apoptotic signalling in the beta cell [2, 3]. Both granzyme B and granzyme A are implicated in beta cell death; however, NOD mice deficient in either granzyme are not protected from diabetes [4, 5], indicating redundancy in the cytotoxic signalling pathways downstream of perforin pore formation. Consequently, we wished to determine whether perforin’s role as an indispensable enabler of granzyme delivery would make it a potential target for pharmacological intervention.

Genetic evidence in support of this notion comes from studies showing that the lifetime incidence of diabetes in perforin-gene-null NOD (NOD.*Pfp*^–/–^) mice is reduced by ~80% (note that *Pfp* is also known as *Prf1*) [6]. These and other studies indicate that perforin-dependent signalling is the dominant (although not exclusive) mechanism through which beta cells are eliminated [7]. Benzenesulfonamide (BZS) compounds developed recently as inhibitors of perforin pore formation [8] can afford protection to a wide variety of tissues targeted by autoreactive or antiviral CD8^+^ T cells in quite diverse pathophysiological scenarios in vivo (discussed below). We therefore investigated whether BZS perforin inhibitors can also protect NOD beta cells from CD8^+^ T cell-mediated killing, both in vitro and in vivo.

## Methods

Please see the electronic supplementary material (ESM) Methods for additional information.

### Mice

Animal studies were conducted at St Vincent’s Institute, approved by the institutional animal ethics committee. Monitoring for diabetes was done by urine glucose measurement (Diastix, Bayer, Leverkusen, Germany), confirmed by two consecutive blood glucose readings >15 mmol/l (Glucose Strips, Roche, Basel, Switzerland).

### Islet isolation

Pancreatic islets were isolated using collagenase P (Roche) and Histopaque-1077 (Sigmaaldrich, Bayswater, Australia), as previously described [9].

### Tetramer staining and magnetic bead-based enrichment

Single-cell spleen suspensions were prepared by mechanical disruption followed by red cell lysis and passage through a 70 μm filter. Islet-specific glucose-6-phosphatase catalytic subunit-related protein (IGRP)_206–214_ (VYLKTNVFL) H2-Kd tetramer (NIH Tetramer Core Facility) staining and enrichment was performed as previously described [9].

### Flow cytometry

Spleen or islet cell suspensions were stained for 30 min at 4°C and analysed on a spectral flow cytometer (Crytek Aurora). Details of antibodies used are given in the ESM Methods.

### In vitro islet killing assay

T cells from NOD8.3 TCR-transgenic mice were stimulated to attain cytotoxicity for use in in vitro killing assays. [^51^Cr]-labelled islets were preincubated with perforin inhibitor SN34960 (10 μmol/l) or diluent (DMSO) for 1 h, then CTLs were added for 16 h.

### In vivo cytotoxicity assay

13- to 15-week-old NOD mice were treated with vehicle or perforin inhibitor (100 mg/kg) via intraperitoneal (i.p.) injection 30 min before the transfer of target cells. Target cells were stained with a high or low concentration of carboxyfluorescein succinimidyl ester (CFSE) and loaded with 10 μmol/l IGRP peptide or no peptide, respectively. In vivo killing assays were performed as previously described [10].

### Treatment of prediabetic mice with SN34960

Wild-type (WT) or NOD.*Pfp*^–/–^ mice received anti-PDL1 antibody by i.p. injection (clone M1H5; JJP Biologics) (250 μg/mouse) on days 1, 3 and 5 and were monitored for diabetes development to 20 days. Perforin inhibitor SN34960 nanosuspension dissolved in PBS was given twice daily by i.p. injection (100 mg/kg, days 3–9 inclusive).

### Statistical analysis

All statistical analysis was performed using GraphPad Prism 8 software (GraphPad, USA), www.graphpad.com. Data in bar graphs are presented as mean ± SD but mean ± SEM is shown for in vitro cytotoxicity assays. *p*<0.05 was considered significant. A Shapiro–Wilk test was performed to determine the normality of the data. A Mann–Whitney test was performed when data were not normally distributed, and a Student’s *t* test was performed for the rest of the data. Multiple comparisons were performed using a one-way ANOVA with a Tukey or Bonferroni post hoc test. Diabetes incidence was compared using a logrank (Mantel–Cox) test.

## Results

**Fig. 1:**
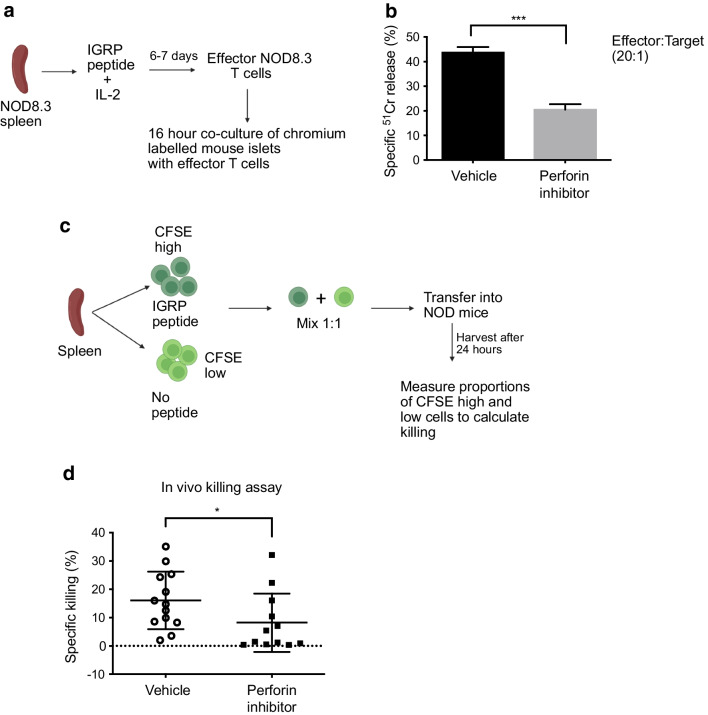
Perforin inhibitor reduces CD8^+^ T cell meditated killing of target beta cells. (**a**) Diagram showing how NOD8.3 T cells were stimulated to acquire cytotoxic capability and the set-up for in vitro cytotoxicity assay with NOD mouse islet target cells. (**b**) Activated CD8^+^ T cells from NOD8.3 mice were incubated with ^51^Cr-labelled NOD mouse islet cells in the presence of perforin inhibitor SN34960 (10 μmol/l) or DMSO diluent (vehicle) over 16 h. Each islet contains ~1000 cells, such that the final effector-to-target ratio was ~20:1. Data represent mean ± SEM from three experiments, unpaired Student’s *t* test ***p<0.001. (**c**) Diagram outlining in vivo cytotoxicity assay with IGRP peptide-labelled target cells. (**d**) Prediabetic NOD mice (12–14 weeks of age) were treated with a single dose of perforin inhibitor (100 mg/kg) or vehicle by i.p. injection and then received a 1:1 mixture of IGRP peptide-loaded and -unloaded splenocytes as targets. 24 h later, spleens were harvested and the percentage killing of the peptide-loaded target cells was calculated, relative to target cells that were not peptide loaded. Pooled data showing mean ± SD of peptide-specific cytotoxicity (*n*=12–13 mice/group), Mann–Whitney test *p<0.05. Schematic illustrations were created using Biorender (www.biorender.com)

Our hypothesis was that small molecule perforin inhibitors can protect beta cells from autoreactive CTLs. For in vitro studies, we used CD8^+^ T cells from TCR-transgenic NOD8.3 mice in which most CD8^+^ T cells recognise a peptide derived from the beta cell antigen IGRP [11]. In vitro stimulation of NOD8.3 T cells with IGRP peptide and IL-2 generates CTLs that recognise and kill primary mouse islet cells [12]. The addition of perforin inhibitor SN34960 to ^51^Cr release cytotoxicity assays reduced the death of primary islet cells by approximately 50% (Fig. 1a, b). Next, we performed an in vivo cytotoxicity assay in which syngeneic donor splenocytes were labelled with IGRP peptide or no peptide [10]. Peptide-positive or -negative targets were marked with different concentrations of CFSE and adoptively transferred in equal number into prediabetic 13- to 15-week-old NOD mice (Fig. 1c). At this age, NOD mice already have established islet inflammation and an increased number of IGRP-specific CD8^+^ T cells in their spleen [9]. Killing of the adoptively transferred IGRP peptide-loaded target cells was significantly reduced in mice that received a single dose of perforin inhibitor by i.p. injection prior to the cell transfer, compared with mice receiving drug diluent only (Fig. 1d).

**Fig. 2:**
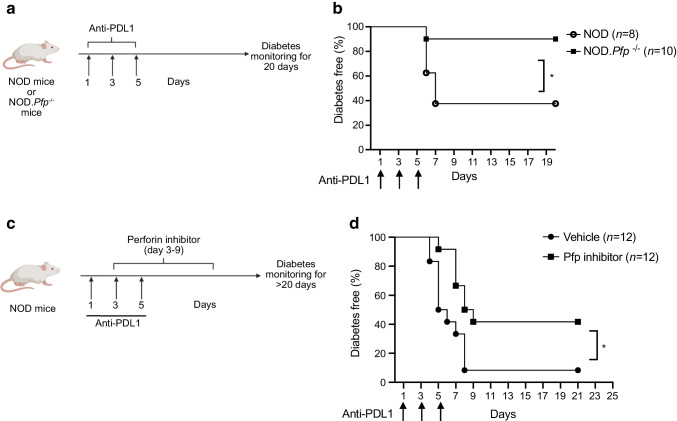
Perforin inhibition protects prediabetic NOD mice from immune checkpoint blockade-induced diabetes. (**a**) Anti-PDL1 treatment schedule and diabetes monitoring scheme. (**b**) Diabetes incidence in WT or NOD.*Pfp*^–/–^ mice after anti-PDL1 antibody (250 μg/mouse per dose) treatment on days 1, 3 and 5 (*n*=8–10 mice/group). (**c**, **d**) Mice were treated with vehicle or perforin inhibitor (100 mg/kg twice daily) by i.p. injection for 7 days starting from day 3 and monitored for diabetes development (*n*=12/group) as detailed in the treatment scheme shown in (**c**). Statistical analysis was done by logrank (Mantel–Cox test), *p<0.05

To determine whether perforin inhibition can also protect the beta cells of NOD mice from autoreactive CD8^+^ T cells in vivo, we used a monoclonal antibody (mAb) to block the immune checkpoint PDL1 on the beta cells, thus accelerating and synchronising disease onset [13]. As a prelude, we determined whether congenital perforin deficiency reduces the incidence of diabetes when PDL1 is inhibited. When WT and perforin-deficient (NOD.*Pfp*^–/–^) mice received three doses of anti-PDL1 mAb via i.p. injection, the incidence of diabetes was reduced from approximately 60% in WT mice to 10% in the perforin-deficient mice (Fig. 2a, b). Consistent with previous studies [6], these results indicated that perforin plays a dominant, but non-exclusive, role in beta cell death in NOD mice. Next, prediabetic WT NOD mice receiving anti-PDL1 antibody were injected with either perforin inhibitor SN34960 or diluent alone by i.p. injection, commencing 2 days after the first dose of anti-PDL1; treatment continued until day 9, that is, for a total of 7 days, and mice were monitored for glycosuria for 20 days. Approximately 91% of diluent-treated mice developed diabetes compared with 58% that received perforin inhibitor across two independent experiments (*n*=12 in each group, *p*<0.05; Fig. 2c, d). Predictably, the impact on disease was less than we observed in NOD.*Pfp*^–/–^ mice because: (1) the CD8^+^ T cells of NOD.*Pfp*^–/–^ mice are completely devoid of perforin activity for the mouse’s entire lifespan; (2) drug treatment was both transient and delayed for 2 days after PDL1 blockade had commenced; and (3) the drug dose was calibrated to produce blood levels that reduce in vivo perforin activity by no more than 70–80% [8].

**Fig. 3:**
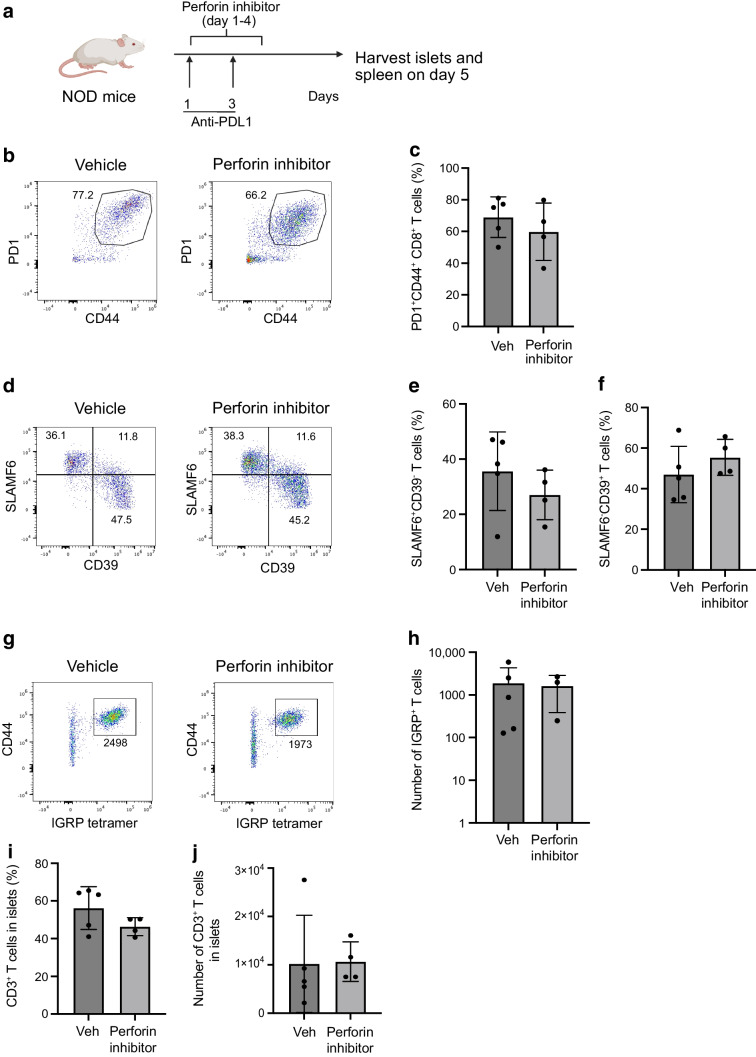
Perforin inhibitor does not impact the activation and differentiation of islet-infiltrating T cells. Prediabetic NOD mice received anti-PDL1 antibody and perforin inhibitor as depicted in (**a**). Pancreatic islets and spleen were harvested and T cells were analysed (*n*=4–5 mice/group). (**b**–**f**) Frequency of (**b**, **c**) PD1^+^CD44^+^ T cells gated on CD8^+^ with stem-like memory (SLAMF6^+^CD39^–^) (**d**, **e**) or effector (SLAMF6^–^CD39^+^) phenotypes (**d**, **f**) among PD1^+^CD44^+^ activated CD8^+^ T cells in the islets. (**g**, **h**) Number of IGRP tetramer^+^ CD8^+^ T cells in the spleen. (**i**, **j**) Numbers and proportions of CD3^+^ T cells in the islets among live lymphocytes. Unpaired Student’s *t* test (not significant)

In NOD mice, anti-PDL1 treatment typically induces activation and expansion of autoreactive T cells [13] and the differentiation of stem-like memory T cells (SLAMF6^+^CD39^–^) into effector T cells (SLAMF6^–^CD39^+^) [14]. To determine whether perforin inhibition had affected these parameters, we characterised the islet-infiltrating T cells into a further cohort of NOD mice that received anti-PDL1 mAb (two doses) and perforin inhibitor (or DMSO diluent) for 4 days (Fig. 3a). Pharmacological perforin inhibition did not alter the proportion of activated PD1^+^CD44^+^ T cells infiltrating the islets (Fig. 3b, c) and had no impact on the SLAMF6^+^CD39^–^ or SLAMF6^–^CD39^+^ memory/effector subsets, compared with control mice (Fig. 3d–f). Similarly, we observed no reduction in the expansion of IGRP-specific T cells in response to anti-PDL1 when perforin was inhibited pharmacologically (Fig. 3g, h). Neither the proportion (Fig. 3i) nor the total number (Fig. 3j) of islet-infiltrating T cells was impacted by perforin inhibitor treatment.

## Discussion

Our study clearly shows that pharmacological perforin inhibition can protect beta cells from destruction by autoreactive CTLs in vitro and in vivo. Using anti-PDL1 mAb to accelerate and synchronise diabetes onset, we first used NOD.*Pfp*^–/–^ mice to confirm genetically that perforin is critical for beta cell destruction. Then, in WT NOD mice, treatment for 7 days with perforin inhibitor SN34960 provided significant protection against diabetes onset. Enhanced beta cell protection was achieved without affecting the proliferation, activation or differentiation of the autoreactive T cells. This is consistent with the drug’s known mechanism of action, which is to bind to perforin only after its exocytic release from the CTL, and with previous studies in mouse models of diverse inflammatory diseases mediated by CD8^+^ CTLs. These include autoimmune cerebral/retinal vasculitis (Susac syndrome) [15], post-viral liver failure [16] and cutaneous leishmaniasis [17]. In the Susac model, single-cell transcriptomic analysis of the autoreactive CTL also confirmed that the activation status and proliferative capacity of the CTL were unchanged by pharmacological perforin inhibition [15].

Despite contributing to autoimmune tissue damage inflicted by CTLs, perforin is also required throughout an entire lifespan for defence against viruses and other intracellular pathogens and for maintaining immune homeostasis [1, 18, 19]. Therefore, despite the positive effects observed in this ‘proof-of-concept’ study, long-term and complete inhibition of perforin might not be advisable in a therapeutic setting. Instead, repeated short treatment cycles combined with other approaches that reduce T cell effector function might prove both effective and safe.

## Supplementary Information

Below is the link to the electronic supplementary material.

ESM (PDF 167 KB)

## Data Availability

All data supporting the conclusions of this study are stored at St Vincent’s Institute of Medical Research or the Peter MacCallum Cancer Centre and are available on request to PMT or JAT.

## Abbreviations

BZS: Benzenesulfonamide
CFSE: Carboxyfluorescein succinimidyl ester
CTL: Cytotoxic T lymphocyte
i.p.: Intraperitoneal
IGRP: Islet-specific glucose-6-phosphatase catalytic subunit-related protein
mAb: Monoclonal antibody
NOD: Non-obese diabetic
NOD.*Pfp*^–/–^: Perforin-gene-null NOD
TCR: T cell receptor
WT: Wild-type
