# Supplementary material for: Pharmacological inhibition of perforin dampens CD8+ T cell-mediated beta cell destruction in autoimmune diabetes in mice
Source: Diabetologia. 2026 Jun 11;69(9):2599–606. doi: 10.1007/s00125-026-06773-8 (PMC13424576; doi:10.1007/s00125-026-06773-8)
Supplement: Supplementary file 1 — ESM (PDF 167 KB) [file 125_2026_6773_MOESM1_ESM.pdf]

Pharmacological inhibition of perforin dampens CD8$^+$ T cell-mediated beta cell destruction in autoimmune diabetes in mice

ESM methods

Mice

All animal studies were conducted at St. Vincent's Institute with approval from the institutional animal ethics committee. NOD/Lt and NOD perforin *gene-null* (NOD.*Pfp*$^{-/-}$) mice were bred and maintained at Bioresources Centre, St. Vincent's Hospital. Female mice were used for all experiments. Mice were monitored for diabetes by urine glucose measurement (Diastix, Bayer, Leverkusen, Germany, 036-6245) and diabetes was confirmed by two consecutive blood glucose readings higher than 15mmol/l using Glucose Strips (Roche, Basel, Switzerland, 07453744020). Block randomisation was used to distribute similar age group NOD mice across treatment groups. Blinding was not carried out for these studies.

Islet isolation

Pancreatic islets were isolated using collagenase P (Roche) and Histopaque-1077 (Sigma Aldrich, Bayswater, Australia, 10771-500ML), as described[1]. Isolated islets were dispersed into single cell suspension using bovine trypsin (Millipore-Sigma, Bayswater, Australia, 65-022-5MU) and 2mmol/l EDTA in PBS. Single cells were washed and incubated in RPMI supplemented with 10% FBS for 1 hour at 37°C before being further processed.

Tetramer staining and magnetic bead-based enrichment

Single cell suspensions from spleen were prepared by mechanical disruption followed by red blood cell lysis and filtering through a 70 µm filter. Cells were resuspended in 0.5% FCS and 4mM EDTA in PBS (MACS buffer) before processing for tetramer staining and magnetic bead enrichment. Magnetic bead based enrichment was performed as described previously[1]. Single cell suspensions were stained with phycoerythrin (PE) labelled $IGRP_{206\text{-}214}$ (VYLKTNVFL) H2-Kd tetramer (Tetramer core, NIH) for 1 hour at 4°C, then washed and stained with anti-PE magnetic beads (Miltenyi Biotech) for 20 minutes at 4°C. Samples were then washed and run on the AutoMACSpro (Miltenyi Biotech) separator to enrich IGRP tetramer+ T cells. The enriched T cells were then stained for cell surface markers by flow cytometry analysis.

Flow cytometry

Spleen or islet single cell suspensions were stained for 30 minutes at 4°C with anti-CD11b (1:200; eFluor450; eBioscience, 48-0012-82), anti-CD11c (1:200; eFluor450; eBioscience, 48-0114-82), anti-Ly6g/Ly6c (1:200; eFluor450; eBioscience, 48-5931-82), anti-CD45R/B220 (1:200; eFluor450; eBioscience, 48-0452-82), anti-CD3 (1:100; V500; BD, 560771), anti-CD4 (1:400; APC-Cy7; BD, 552051), anti-CD8 (1:300; BV711; Biolegend, 100759), anti-CD44 (1:400; PE-Cy7; Biolegend, 103030), anti-PD1 (1:300; BV605; Biolegend, 135220), anti-Slamf6 (1:100; FITC; Miltenyi, 130-118-597), anti-TIGIT (1:300; PE Dazzle 594; Biolegend, 142110), anti-CD226 (1:300; BV785; Biolegend, 133611). B220, CD11c, CD11b and Ly6G were used for gating out non-T cells and CD3 was used to identify T cells.

In vitro islet killing assay

T cells from NOD8.3 TCR transgenic mice were stimulated into cytotoxic T cells and used in *in vitro* killing assays as described [2], with minor modifications. Briefly, whole islets from 4-6 week NOD mice were loaded with 150 µCi [^{51}Cr] sodium chromate (PerkinElmer, Shelton, Connecticut, USA, NEZ030S005MC) for 120 min, washed and resuspended in complete RPMI-1640. Islets (10/well, uniform shape and size ~ 10,000 cells/well) were incubated with T-cells in triplicate for 16 h at 37°C in 96-well plates. The wells also contained perforin inhibitor SN34960 (10µmol/l) or diluent (DMSO) control. Medium alone or 2% Triton X-100 was added to targets for determination of spontaneous and total cell lysis, respectively. The radioactivity of harvested supernatant was measured using a gamma counter (PerkinElmer). Specific ^{51}Cr release was calculated: percent lysis = (test counts per minute − spontaneous counts per minute)/(total counts per minute − spontaneous counts per minute) × 100.

In vivo cytotoxicity assay

Splenocytes from 4-6 week old NOD mice were used as target cells. Splenocytes were either pulsed with 10µmol/l IGRP 206-214 peptide and labeled with 5µmol/l CFSE (CFSE hi), while control cells received no peptide but were labeled with 0.5µmol/l CFSE (CFSE lo). CFSE hi and CFSE lo cells were mixed in 1:1 ratio and a total of 10^7 cells/recipient were transferred i.v. into 13-15 week old NOD mice. The mice were treated with vehicle or perforin inhibitor (100mg/kg) i/p 30 minutes before transfer of target cells. NOD-PI mice, which do not have IGRP specific CD8$^+$ T cells, were used as negative control recipients to calculate relative killing. After 24 hr, spleens of recipient mice were harvested. Single cell suspensions were prepared and analysis of CFSE hi and CFSE lo cells was performed on a BD LSRFortessa. Percent specific killing was calculated as: $(1-r_{NOD-PI}/r_{NOD}) \times 100$, where r= % CFSE hi / % CFSE lo [3].

[1] Chee J, Ko HJ, Skowera A, et al. (2014) Effector-memory T cells develop in islets and report islet pathology in type 1 diabetes. J Immunol 192(2): 572–580. 10.4049/jimmunol.1302100
[2] Dudek NL, Thomas HE, Mariana L, et al. (2006) Cytotoxic T-cells from T-cell receptor transgenic NOD8.3 mice destroy beta-cells via the perforin and Fas pathways. Diabetes 55(9): 2412–2418. 10.2337/db06-0109
[3] Ko HJ, Chee J, Sutherland RM, et al. (2014) Functional cytotoxic T lymphocytes against IGRP206-214 predict diabetes in the non-obese diabetic mouse. Immunol Cell Biol 92(7): 640–644. 10.1038/icb.2014.29
